# Supplementary material for: Improvements to the Rice Genome Annotation Through Large-Scale Analysis of RNA-Seq and Proteomics Data Sets
Source: Mol Cell Proteomics. 2018 Oct 5;18(1):86–98. doi: 10.1074/mcp.RA118.000832 (PMC6317475; doi:10.1074/mcp.RA118.000832)
Supplement: supplementary File S1 [file RA118.000832_index.html]

Supplement to Improvements to the rice genome annotation through large-scale analysis of RNA-Seq and proteomics datasets | Molecular & Cellular Proteomics

## Supplemental Data

- rice\_proteogenomics\_non\_novel\_peptides.pro.bed - All non-novel (i.e. matching official annotations) peptides identified at 1% PSM FDR in proBed format, suitable for genome visualisation or opening in Spreadsheet software.
- rice\_proteogenomics\_novel\_peptides.pro.bed - All final novel peptides identified, passing all filtration steps in proBed format, suitable for genome visualisation or opening in Spreadsheet software.
- Supplementary file 1 - Details of search results, genome mapping for novel peptides, filtration performed, data set sources and search parameters.
- Supplementary Results - Supplementary results on RNA Seq genome mapping, coding potential and BLAST.
