# Supplementary material for: Improvements to the Rice Genome Annotation Through Large-Scale Analysis of RNA-Seq and Proteomics Data Sets
Source: Mol Cell Proteomics. 2018 Oct 5;18(1):86–98. doi: 10.1074/mcp.RA118.000832 (PMC6317475; doi:10.1074/mcp.RA118.000832)
Supplement: supplementary File S1 [file 137847_1_supp_195239_p7btfm.docx]

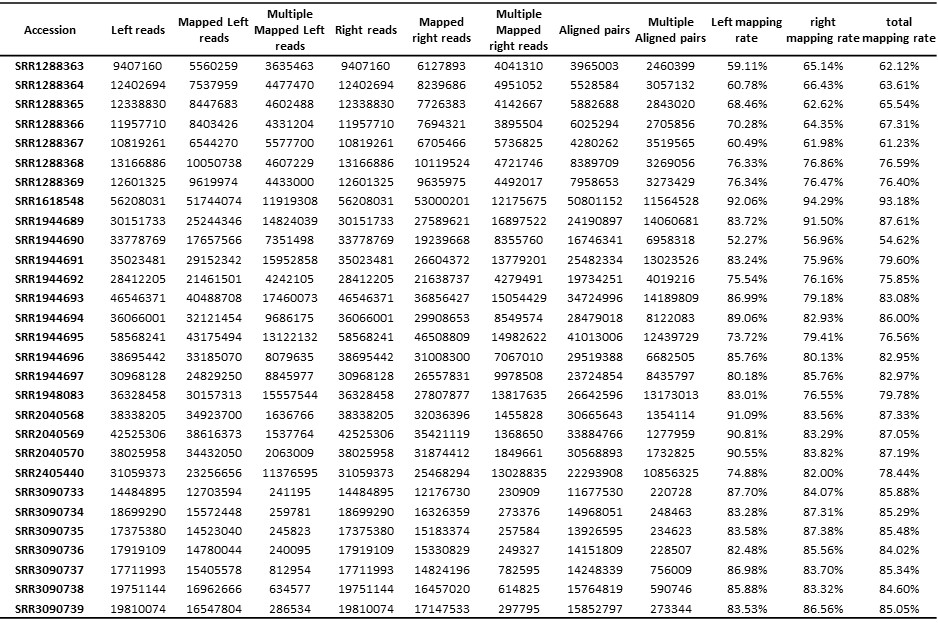


**Supplementary Table 1** Overall mapping rates of the RNA-Seq data from different data sets against the rice annotation IRGSP-1.0.30.


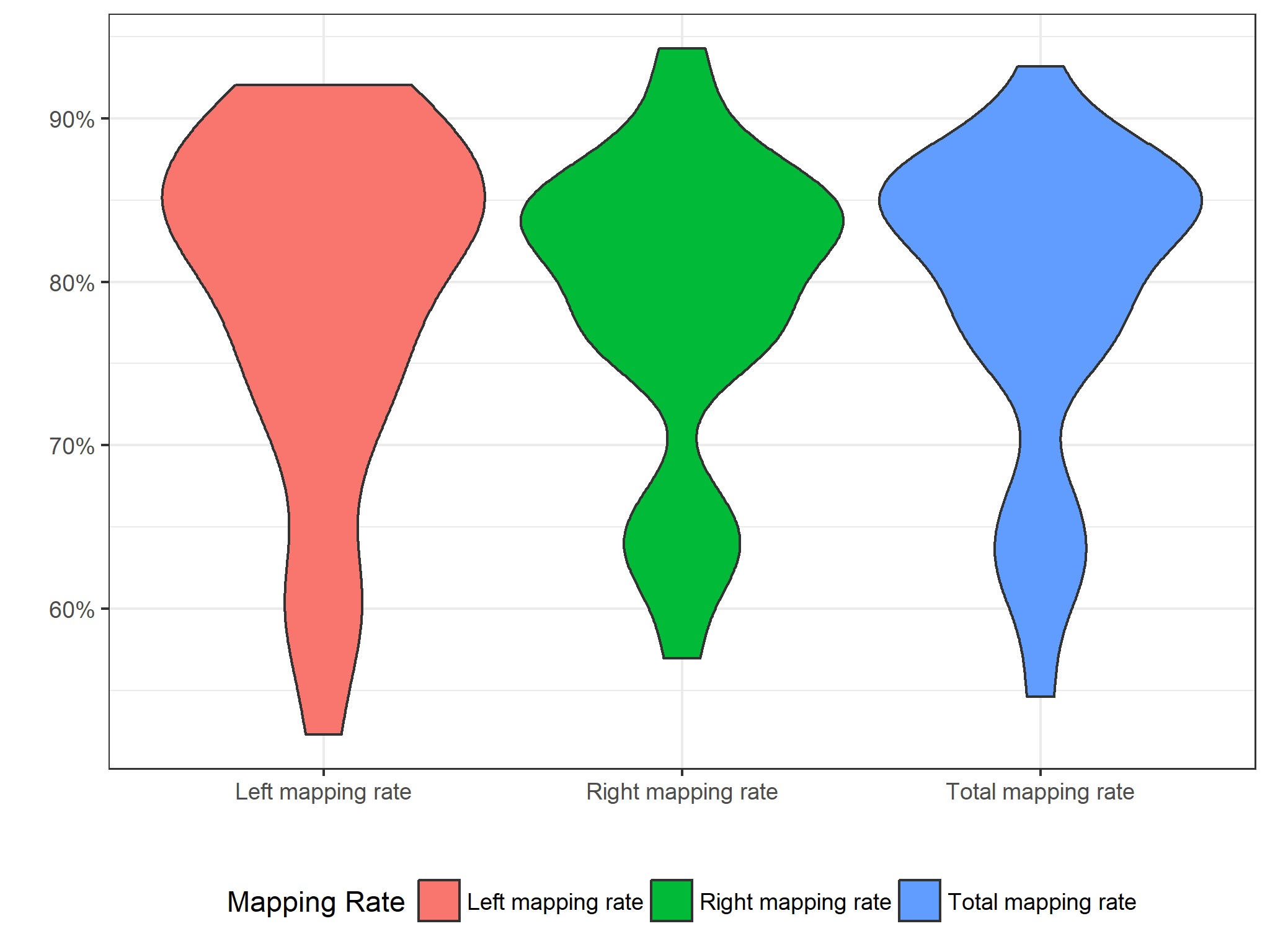


**Supplementary Figure 1. The distribution of mapping rates for the RNA-Seq data.** A violin plot to display an overview of the mapping rate for left end reads (red), right end reads (green) and total (blue). The density shows the frequency of mapping rate across different data sets. There is an enrichment around 80% to 90% which indicates a generally high mapping rate of the RNA-Seq data.


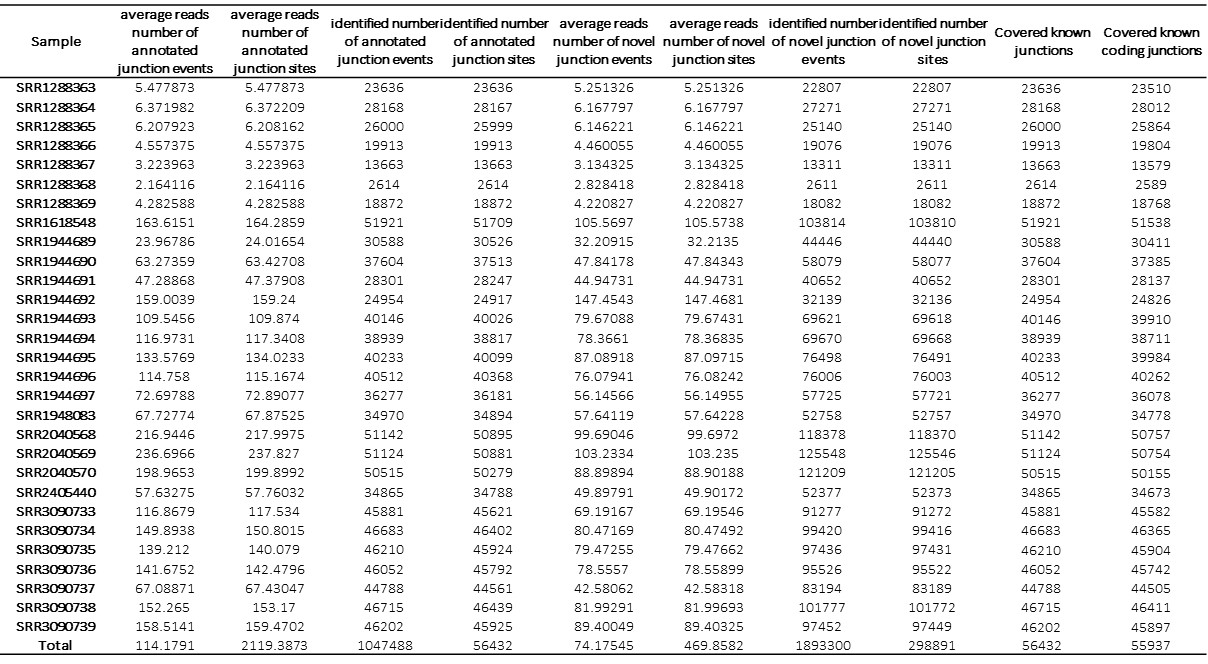


**Supplementary Table 3.** Statistics on the creation of the novel junction database from the different source RNA-Seq data sets.

**Supplementary Figure 2.** Different classes of transcripts assembled by cufflinks. The bar plot shows the transcript count within the different classes and their proportion within the total transcripts. The class code was marked at the right of the bar plot, briefly, class code “=” means the assembled transcript was a complete match of the exons; class code “j” means potentially novel isoform (fragment): at least one splice junction is shared with a reference transcript; class code “o” means generic exonic overlap with a reference transcript; class code “u” means Unknown, intergenic transcript; class code “x” means exonic overlap with reference on the opposite strand; class code “s” means an intron of the transfrag overlaps a reference intron on the opposite strand (likely due to read mapping errors).


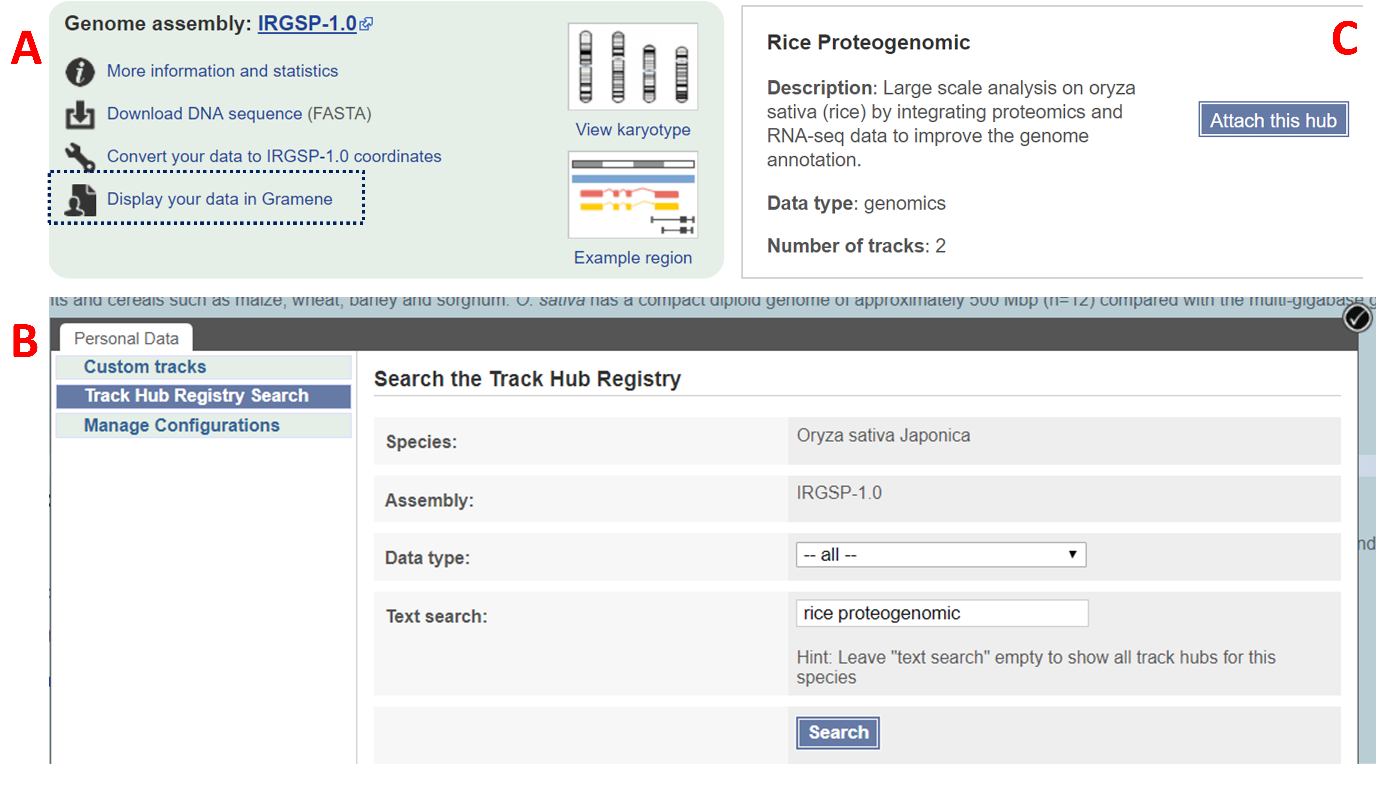


**Supplementary Figure 3** The process for visualising the results of this study via Track Hubs: A) visit <http://ensembl.gramene.org/Oryza_sativa/Info/Index>, and click “Display your data in Gramene”; B) search “rice proteogenomic” in “Track Hub Registry Search”; C) our track hub will be displayed.

**Configuring the visible track hubs**

For the hyperlinks to display our results as tracks, users must first visit the Gramene oryza sativa website at <http://ensembl.gramene.org/Oryza_sativa/Info/Index> as shown in supplementary figure 3A. Click “Display your data in Gramene” and in the pop-up window (supplementary figure 3B) click “Track Hub Registry Search”. In the text search box, use the keyword ‘Rice Proteogenomic’ to search for our track hub. Once you find our project as shown in supplementary figure 3C, click “Attach this hub”. You will get the message “Your hub attached successfully”. Now the *Rice Proteogenomic* hub is configured as external data. Through all above steps, the user will be able to see the results of novel and non-novel peptides tracks of each link from the SupplementaryFile1.xlsx.


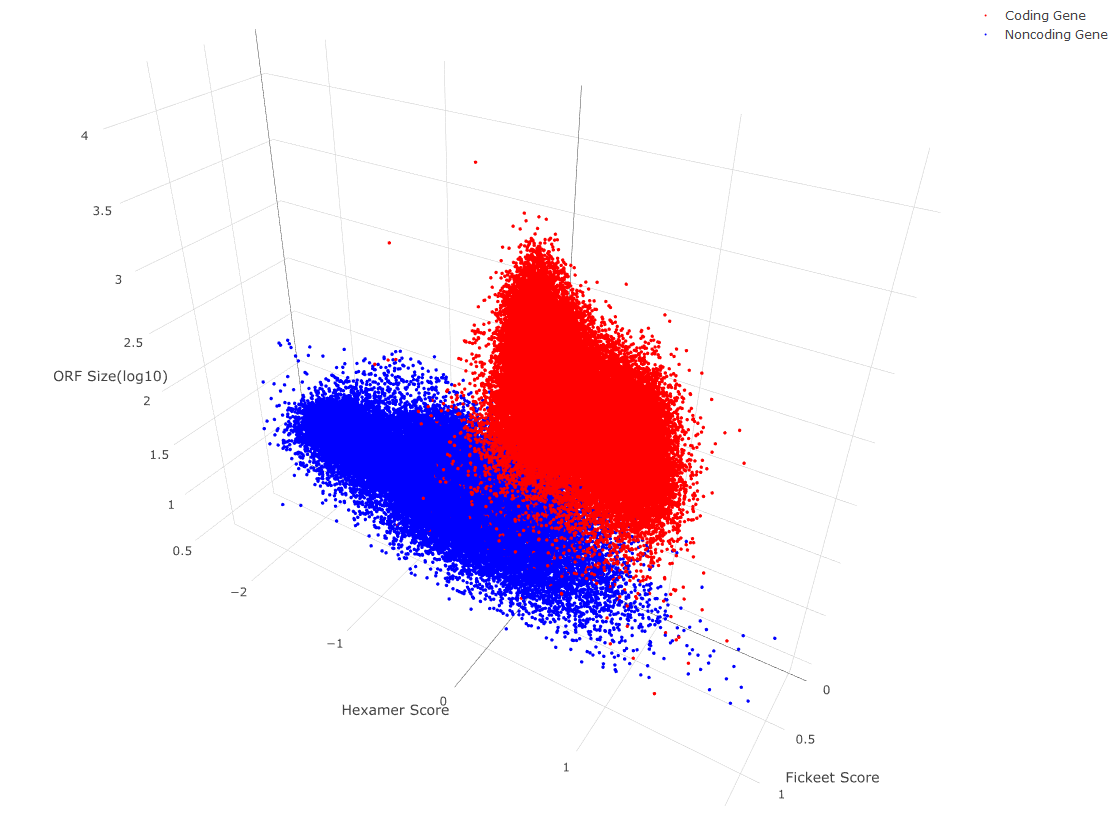


**Supplementary Figure 4.** Training data for calculating Coding potential. A 10-fold cross validation (randomly 4500 out of 42,132 coding transcripts and randomly 4500 out of 53,250 noncoding transcripts) was conducted to build the model. The combinatorial effects of the major features (ORF size, Fickeet score and Hexamer score) of total 42,132 coding transcripts and 53,250 noncoding transcripts was calculated and shown in the figure. There is a clear separation of the two sets.


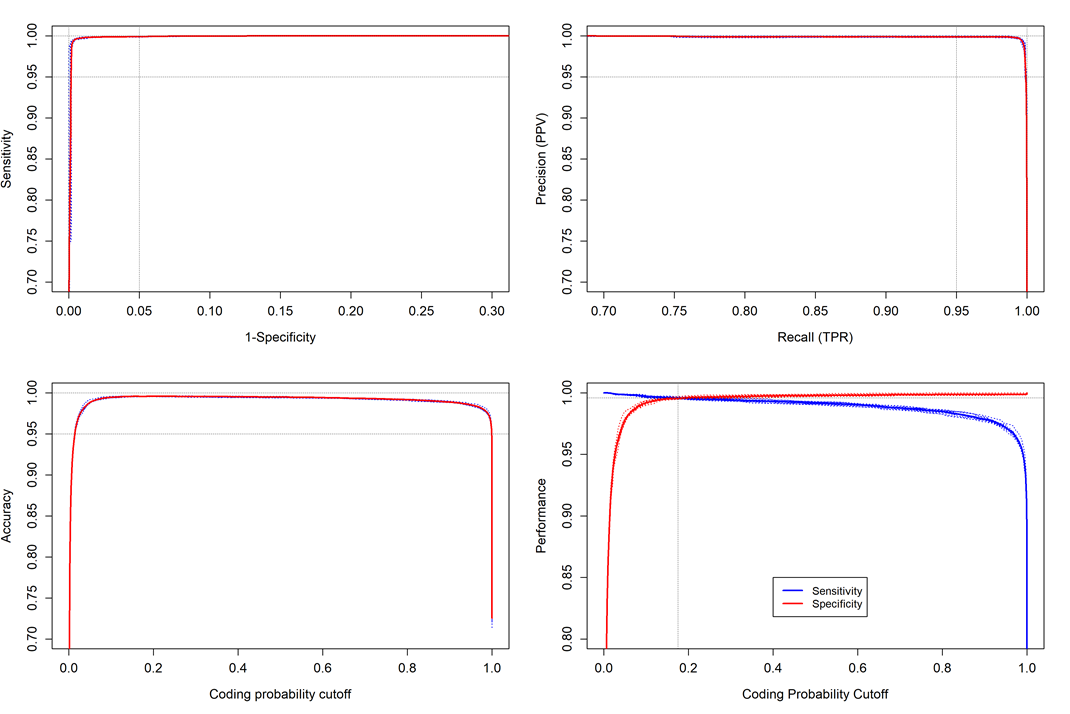


**Supplementary Figure 5. Finding the threshold of Coding potential.** The ROC curve and precision-recall curve of 10-fold cross validation. The optimum coding probability cut-off was equal to 0.175 by finding the intersection of sensitivity curve and specificity curve (Sensitivity=Specificity=0.996).


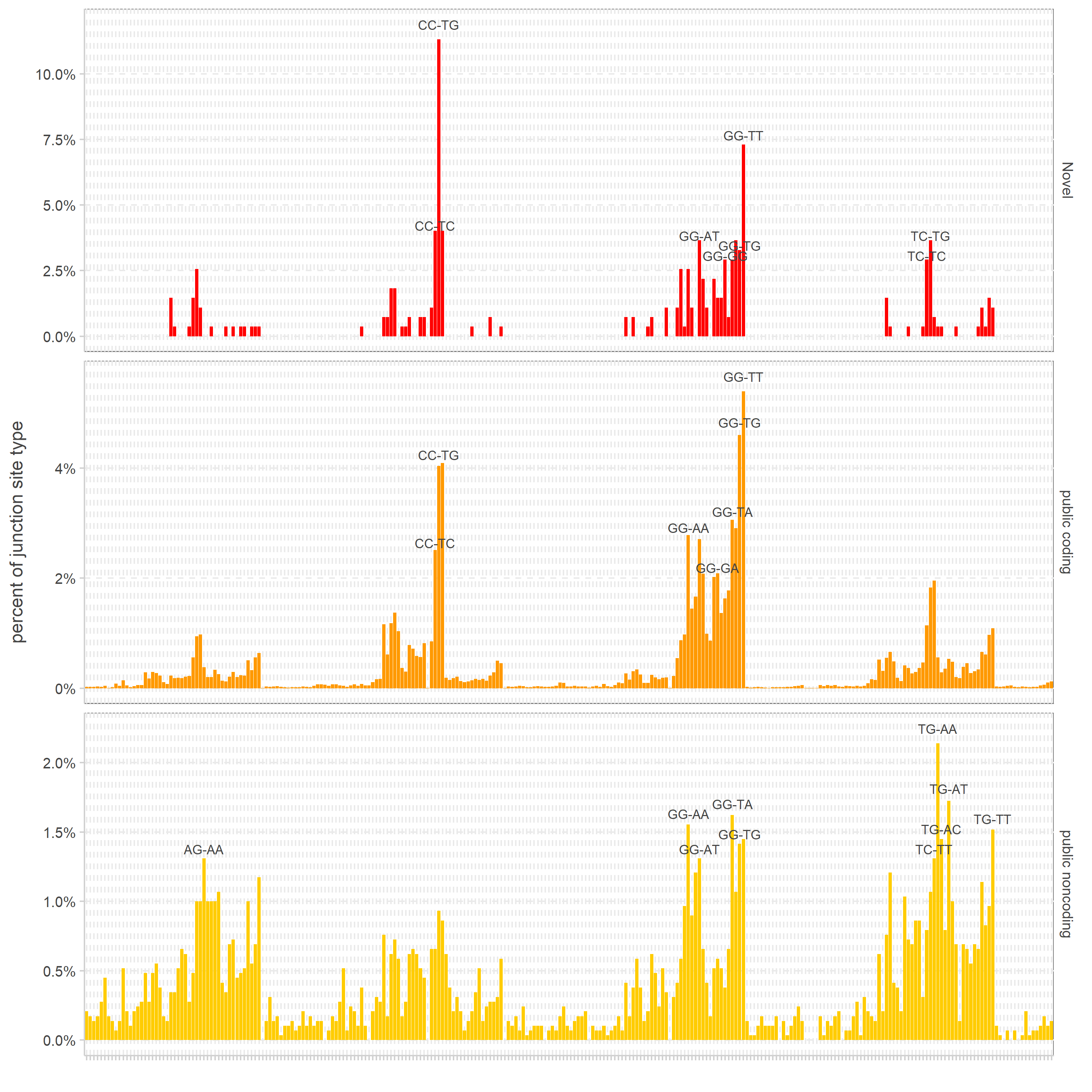


**Supplementary Figure 6.** The percentage of the four bases (two 5’ and two 3’) participating in splice junctions for novel junctions supported by FNPs (top panel), known junctions from official coding genes (middle panel) and known junctions from the official noncoding genes (bottom panel). The splice junctions are in alphabetical order on the x-axis, running left to right i.e. starting with AA-AA, and terminating with TT-TT. The top 10 most frequent splice sites in each kind of junctions are marked out.


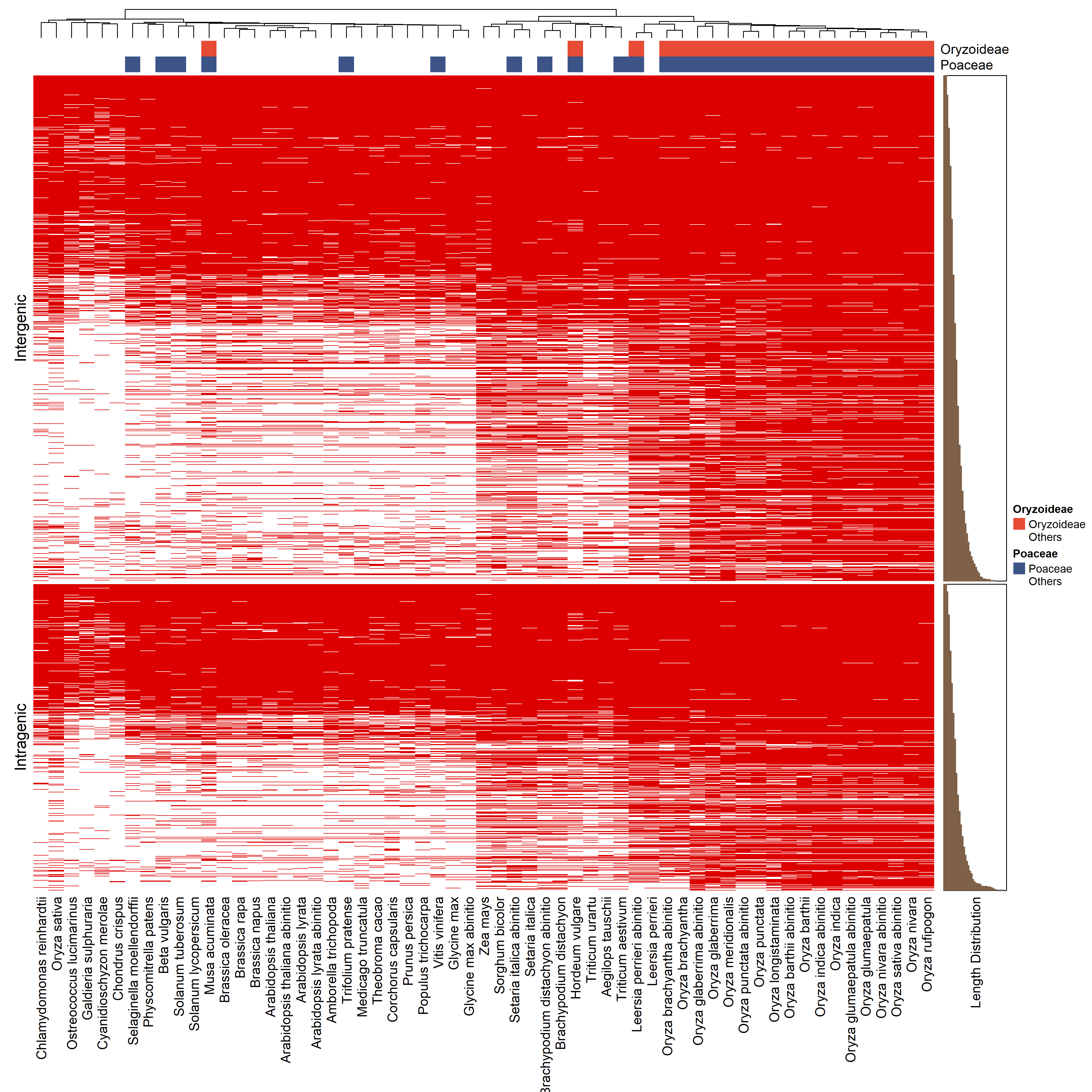


**Supplementary Figure 7.** The heatmap represents hierarchical analysis of the final novel peptides mapped against the proteins encoded by the 44 plant genomes from Ensembl (red = positive match, white = no match from BLASTp, *allowing one gap and two mismatches*). The novel peptides are divided into two groups, intergenic (upper panel) and intragenic (lower panel), and are ranked by peptide length for hierarchical analysis

**References**

1. Kyndt, T., Denil, S., Bauters, L., Van Criekinge, W. and De Meyer, T. (2014) Systemic suppression of the shoot metabolism upon rice root nematode infection. *PloS one*, 10.1371/journal.pone.0106858.
